# Adding hydrogen atoms to molecular models via fragment superimposition

**DOI:** 10.1186/s13015-022-00215-x

**Published:** 2022-03-29

**Authors:** Patrick Kunzmann, Jacob Marcel Anter, Kay Hamacher

**Affiliations:** Computational Biology and Simulation, Technical University of Darmstadt, Schnittspahnstraße 2, 64287 Darmstadt, Germany

**Keywords:** Open source, Python, Structural biology, Structural biophysics

## Abstract

**Background:**

Most experimentally determined structures of biomolecules lack annotated hydrogen positions due to their low electron density. However, thorough structure analysis and simulations require knowledge about the positions of hydrogen atoms. Existing methods for their prediction are either limited to a certain range of molecules or only work effectively on small compounds.

**Results:**

We present a novel algorithm that compiles fragments of molecules with known hydrogen atom positions into a library. Using this library the method is able to predict hydrogen positions for molecules with similar moieties. We show that the method is able to accurately assign hydrogen atoms to most organic compounds including biomacromolecules, if a sufficiently large library is used.

**Conclusions:**

We bundled the algorithm into the open-source Python package and command line program Hydride. Since usually no additional parametrization is necessary for the problem at hand, the software works out-of-box for a wide range of molecular systems usually within a few seconds of computation time. Hence, we believe that Hydride could be a valuable tool for structural biologists and biophysicists alike.

**Supplementary Information:**

The online version contains supplementary material available at 10.1186/s13015-022-00215-x.

## Background

Structural knowledge in biomolecules needs to take hydrogen atoms into account. They are crucial for detection of hydrogen bonds and molecular dynamics simulations.

Currently only $\sim \!16\,\%$ of all Protein Data Bank (PDB) [1] entries (28,971 out of 181,847) contain hydrogen atoms. Furthermore and augmenting the problem, some simulation and molecular docking methods omit hydrogen in their molecule representation [2–4] and hence also in the output structure files.

Consequently, most molecular structure files need to be processed, i.e. hydrogen atoms need to be added, before further analysis or simulations can be performed. The existing methods for this purpose are often bundled into large molecular dynamics simulation packages like Gromacs [5] (pdb2gmx) and CHARMM [6] (HBUILD) or are available as single programs like REDUCE [7], OpenBabel [8] or HAAD [9]. However, most of these programs predict hydrogen positions based on force fields that were parametrized only for a very limited number of different molecules. An exception is OpenBabel, which is not restricted to a set of parametrized compounds, but focuses on small molecules.

Here we describe a novel method for addition of hydrogen atoms to molecular models in general: from large macromolecules to small ligands. The underlying algorithm leverages the geometric information about hydrogen atoms from a library of fragments built from reference molecules containing hydrogen atoms. Based on this information the method is able to accurately predict hydrogen positions for molecules containing equivalent groups. Based on this algorithm, we developed a Python implementation that provides both, a user-friendly *command line interface* (CLI) and a more flexible Python API based on the bioinformatics library Biotite [10].

## Implementation

The aim of the presented algorithm is to add hydrogen atoms to a molecular model, where these are missing. This molecular model will be called *target molecule*, though it may also constitute a model with multiple molecules. The algorithm expects that all atoms except hydrogen, so called *heavy atoms*, are present and accurately placed in the target molecule. The algorithm performs the prediction of hydrogen atoms in two steps, where the second one is optional: hydrogen addition and relaxation.

### Initial hydrogen addition

#### Library compilation

**Fig. 1:**
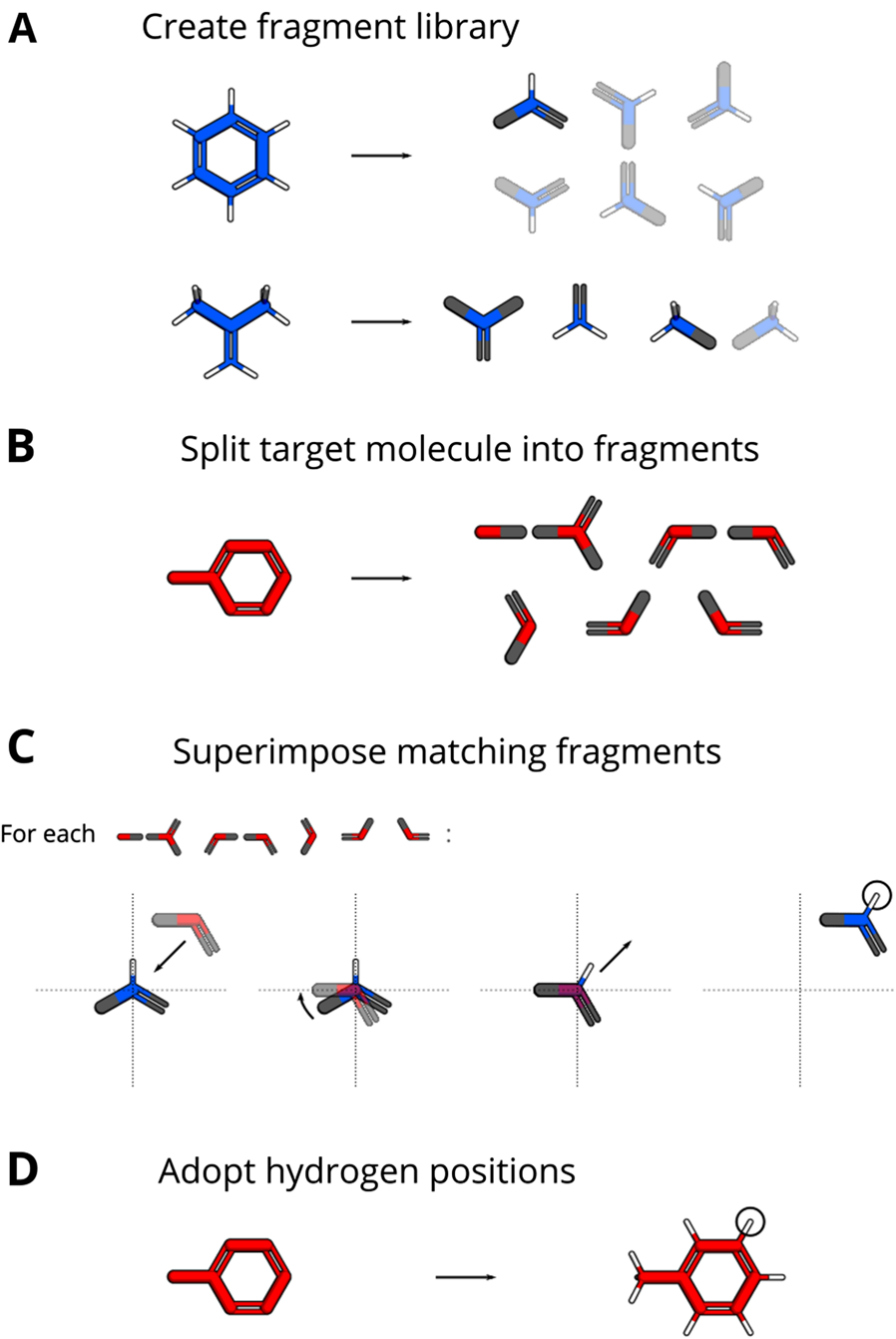
Hydrogen addition algorithm in a nutshell. This figure displays how hydrogen atoms are added to toluene (target) from information about benzene and isobutylene (reference). **A** The reference molecules are split into fragments. The central atom is shown in blue, connected heavy atoms in gray and hydrogen atoms in white. The opaque fragments are added to the fragment library, the transparent ones are ignored due to being duplicates. **B** The target molecule is split into fragments. The central atom is shown in red and connected heavy atoms in gray. **C** For each target fragment the matching library fragment is superimposed. The central atom for both fragments are positioned in the coordinate origin, the library fragment is rotated to achieve congruence and the library fragment is translated to the original position of the target fragment. The resulting coordinates of the hydrogen atom(s) (encircled) are taken. **D** The obtained hydrogen positions from the previous step are adopted for the target molecule

The addition of hydrogen atoms is based on known molecular geometries of reference molecules. For this purpose the reference molecules are compiled into a *fragment library* (Fig. 1A): Each reference molecule is split into *fragments*, one for each heavy atom in the molecule. Each fragment consists of

- the element, formal charge and coordinates of the central heavy atom,
- the coordinates of the bonded hydrogen atoms,
- the coordinates of the bonded heavy atoms and the order of the bonds connecting them and
- the chirality of the fragment, if applicable.

These fragments are stored in the aforementioned fragment library, a data structure that maps the combination of a fragment’s

- central atom element,
- central atom formal charge,
- chirality and
- order of bonds with connected heavy atoms

(called *library key* from now on) to

the coordinates of heavy atoms connected to the central atom and

the coordinates of hydrogen atoms connected to the central atom.

**Fig. 2:**
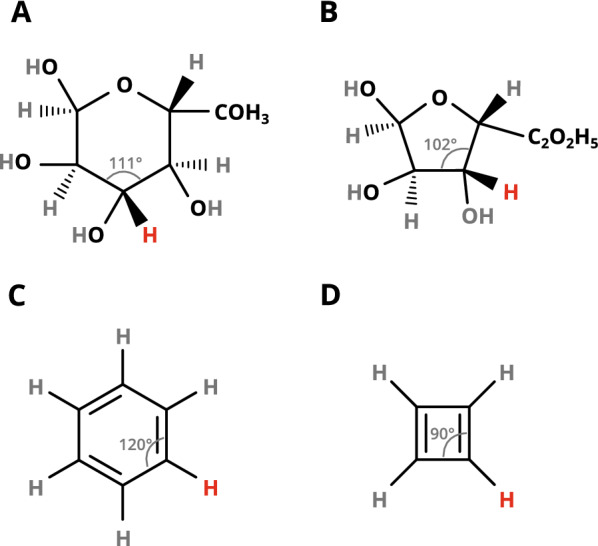
Molecule pairs with different geometries for the same library key. **A** $\alpha$-d-Glucopyranose. **B** $\alpha$-d-Glucofuranose. **C** Benzene. **D** Cyclobutadiene. Each pair of molecules **A+B** and **C+D** has the same library key for the carbon atom bonded to the hydrogen atom highlighted in red, but the geometry, signified by the bond angle, is different

The coordinates of the fragment’s central atom are not saved, as the coordinates of the fragment are always translated to position the central atom in the coordinate origin. Duplicate library keys are ignored and hence will not be part of the fragment library: If two fragments with equal library keys are added to the library, the library will contain the coordinates of the latter one. Hence, this algorithm does not distinguish between the different possible geometries of heavy atoms for the same library key, as observed in cyclic compounds (Fig. 2). However, this does not affect the hydrogen positioning in a sufficiently significant manner (as discussed later) to justify a more time-consuming step for identification of the fragment with the most suitable heavy atom geometry.

Nitrogen as a central atom requires special handling, due to its ability to form partial double bonds using its lone electron pair. This means, that although a fragment with nitrogen as a central atom only possesses single bonds, such a partial double bond still induces a planar conformation in contrast to a tetragonal conformation. To obtain the correct geometry in this special case, a separate bond order is used for partial double bonds.

#### Target molecule fragmentation

The target molecule is split into fragments in a similar fashion (Fig. 1B). But in contrast to the molecules for the fragment library these created fragments naturally do not contain hydrogen atoms.

For each target molecule fragment (*target fragment* in short), the matching fragment from the fragment library (*library fragment* in short) is selected, i.e. the fragment with the same library key. Although the target fragment has no hydrogen atoms, the corresponding library key can still be created, because the hydrogen atoms are not part of the library key.

If the library does not contain a match for a target molecule fragment, the algorithm is unable to assign hydrogen atoms to this central atom. Hence, it is desirable to have a large fragment library to cover a broad range of different fragments. For our implementation we used all molecules from the *Chemical Component Dictionary* (CCD) [11] for the fragment library compilation. This guarantees, that hydrogen atoms can be added to all molecules appearing in the PDB. Furthermore, most other organic molecules are implicitly supported, because they share the same fragments as the organic molecules added from the CCD.

#### Fragment superimposition

Now the library fragment is superimposed onto the target fragment (Fig. 1C). For this purpose, the target fragment coordinates are translated so that the central atom lies in the coordinate origin. The central atom of the library fragment already lies in the origin. Then the library fragment is superimposed onto the target fragment by rotation about the coordinate origin [12, 13]. The two fragments probably do not overlap perfectly, but the superimposition minimizes the *root-mean-square deviation* between the fragments. In the final step the library fragment is translated back to the original position of the target fragment by applying the reversed translation vector. The hydrogen coordinates of the transformed library fragment are the desired coordinates for the target fragment.

After this procedure is finished for each target fragment, the obtained hydrogen positions are adopted by the target molecule (Fig. 1D).

### Relaxation of terminal groups

After initial placement of hydrogen atoms, most of their positions should be accurate, as they are constrained by the position of the respective bonded heavy atom, since the bond lengths and angles are (approximately) constant. However, there are exceptions: Terminal heavy atoms connected with a single bond to the remaining molecule, e.g. a hydroxy or methyl group, have no unambiguous hydrogen positions, as they are able to rotate about this single bond. Hence, the positions of hydrogen atoms bonded to these terminal heavy atoms are relaxed in a second step.

#### Energy function

The energy function *V* required for the relaxation is based on the non-bonded interaction terms of the *Universal Force Field* (UFF) [14]. The interaction terms comprise an electrostatic $V_\text {el}$ and a *Lennard-Jones* $V_\text {LJ}$ term. For the position vectors $\vec {r}_i$ and $\vec {r}_j$ of two atoms *i* and *j* the contribution to the energy function is

1 $$\begin{array}{ll} V_(\vec {r}_i, \vec {r}_j)= V_\text {el}(\vec {r}_i, \vec {r}_j) + V_\text {LJ}(\vec {r}_i, \vec {r}_j) \\ V_\text {el}(\vec {r}_i, \vec {r}_j)= 332.067 \frac{q_i q_j}{D_{ij}} \\ V_\text {LJ}(\vec {r}_i, \vec {r}_j)= \epsilon _{ij} \left( \left( \frac{\delta _{ij}}{D_{ij}}\right) ^{12} - 2\left( \frac{\delta _{ij}}{D_{ij}}\right) ^6 \right) . \end{array}$$

$D_{ij}$ is the euclidean distance between the atoms *i* and *j*.

2 $$\begin{aligned} D_{ij} = | \vec {r}_j - \vec {r}_i |. \end{aligned}$$

$\epsilon _{ij}$ and $\delta _{ij}$ are the well depth and optimal distance between these atoms, respectively, and are calculated as

3 $$\begin{aligned} \begin{aligned} \epsilon _{ij}&= \sqrt{ \epsilon _i \epsilon _j}, \\ \delta _{ij}&= \frac{\delta _i + \delta _j}{2}. \end{aligned} \end{aligned}$$

$\epsilon _{i/j}$ and $\delta _{i/j}$ are taken from the UFF and solely depend on the chemical element of the respective atom. To obtain more accurate distances for hydrogen bonds, $\delta _{ij}$ is multiplied with 0.79 for potential hydrogen bond acceptor–donor pairs [15]. By default, the charges *q* are calculated via the PEOE method [16] implemented in Biotite [10].

The units are given in (kcal/mol) for energies and Å for lengths. Charges are given in multiples of the elementary charge.

Interactions are calculated between all pairs of rotatable hydrogen atoms and all other atoms within a defined cutoff distance of 10 Å. All other interaction pairs do not need to be considered, as their distances to each other are not altered during the course of relaxation.

#### Relaxation algorithm

Based on this energy function, the applicable hydrogen atoms are iteratively rotated about the bond of the terminal heavy atom. However, if the terminal heavy atom is bonded via a (partial) double bond to the rest of the molecule, free rotation is prohibited. For imine groups, as they appear e.g. in arginine, two hydrogen conformations are still possible though. Due to these discrete values a continuous optimizer cannot be employed. Hence, our method uses a variant of the *hill climbing* algorithm, that aims to reach local minimum of the energy function *V*.

Let $\phi _1 \ldots \phi _n$ be the dihedral angles of the rotatable terminal bonds $1 \ldots n$. Each $\phi _k$ affects the positions $\vec {r}_p \ldots \vec {r}_q$ of the hydrogen atoms bonded to the corresponding heavy atom.

In each iteration the dihedral angles of all rotatable bonds are altered by a an angle increment $\Delta \phi$ in alternating direction. $\Delta \phi$ is small (by default 10$^\circ$) or 180$^\circ$ for freely rotatable bonds and imine groups, respectively. Let $\phi _1^* \ldots \phi _n^*$ be these updated angles. Let $\vec {r}_p^* \ldots \vec {r}_q^*$ be the new positions resulting from the new angle $\phi _k^*$.

For each rotatable bond *k*, the energy difference with respect to the change in $\phi _k$, called $\Delta V^*$, is determined by

4 $$\begin{aligned} \Delta V^*(k) = \sum _{a=p}^q \sum _b^\text {all} \left[ V(\vec {r}_a^*, \vec {r}_b) - V(\vec {r}_a, \vec {r}_b) \right] . \end{aligned}$$

Thus, all interaction terms are evaluated that involve the atoms $p \ldots q$ affected by the rotatable bond *k*. For each interaction term, the energy difference between the positions before and after the isolated update of $\phi _k$ is calculated. $\Delta V^*$ is the sum of these energy differences.

If $\Delta V^*(k)$ is negative, the new dihedral angle for bond *k* is preferable, as it leads to a lower energy. Hence, $\phi _k^*$ is accepted and used as the new $\phi _k$ in the next iteration. Otherwise, it is rejected and the next iteration uses the $\phi _k$ from the previous iteration.

When within two subsequent iterations no $\phi _k^*$ is accepted for any *k*, the energy has reached the local minimum and the algorithm has finished.

As alternative variant we tested a *multistart* approach: Initial random values were assigend to the rotatable angles $\phi _1 \ldots \phi _n$ before the described algorithm was executed. This process was repeated multiple times and the conformation with the lowest energy *V* was accepted. However, we found no noteworthy accuracy improvements over the original method.

### Formal charge calculation

Commonly, input structures do not contain atoms with physiological formal charges, but most charges are given as neutral instead. Consequently, the described algorithm would treat acidic groups as protonated and basic groups as deprotonated. To mitigate this issue, our algorithm optionally recalculates charges for atoms in amino acids based on the tabulated $pK_a$ value of the respective free amino acid [17] and a user-provided *pH* value.

### Atom order and naming

If the given molecular model contains multiple residues, the associated hydrogen atoms are placed behind the heavy atoms from the same residue in the list of atoms and consequently also in the written output file.

For common residues appearing in macromolecular structures, including amino acids and canonical nucleotides, the hydrogen atoms are named according to the nomenclature from the CCD, e.g. the hydrogen atom for ’CA’ is named ’HA’. For all other cases a reasonable atom naming scheme is picked based on the bonded heavy atom name.

**Fig. 3:**
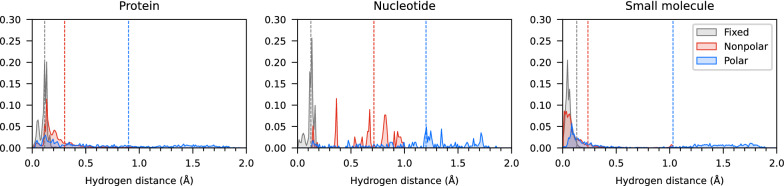
Accuracy of hydrogen position prediction. This figure shows histograms of distances between the reference and predicted hydrogen position for each dataset and group. The dashed lines represent the RMSD for the distances in each group. The histograms for each group are normed

### Python package

Based on the described algorithm we developed the Python package Hydride. Besides hydrogen addition based on the CCD reference dataset, it also allows the user to add extra reference molecules to the fragment library to achieve a higher hydrogen prediction accuracy for these molecules. The API of the package builds upon data types from the bioinformatics library Biotite [10], harnessing its support for different structure file formats, including PDB, mmCIF, MMTF [18], MOL and SDF [19]. Furthermore, Biotite is used for calculation for distances and displacement vectors between atoms, which optionally take periodic boundary conditions into account. To compensate for the relatively low computation speed of the programming language, the time consuming task of the fragment superimposition and relaxation is vectorized via NumPy [20] and accelerated using a C-extension written in Cython [21], respectively. This package can be installed via the pip and Conda package managers.

## Results and discussion

### Accuracy of predicted hydrogen positions

We validated the hydrogen prediction algorithm against a dataset of high resolution protein and nucleic acid structure data. We chose the protein structures used by Li et al. [9] for comparison with the HAAD algorithm. For the nucleic acid dataset we selected all nucleic acid structures with a resolution $\le 1.0\,$ Å. Furthermore, we assembled a dataset containing 5000 random small molecule structures from the *PubChem* database [22], to assess whether the algorithm is capable of correct hydrogen assignment to a wide range of organic molecules, even if they are not part of the fragment library.

For the validation we removed all hydrogen atoms from the respective reference structure and added them back via Hydride. Then we measured the distance between each predicted hydrogen atom and the respective original hydrogen atom in the reference structure.

Figure 3 shows the distribution of the measured distances for each dataset. All hydrogen atoms are further divided into three groups, depending on whether they are

- fixed in their position, since there is no rotational freedom, or
- rotatable and part of nonpolar or
- polar terminal group.

The scripts and Snakemake workflow [23] to reproduce this benchmark are available in Additional file 1.

#### Protein structures

On average our algorithm achieved an RMSD $= 0.247\,$ Å, that is similar to HBUILD (RMSD $= 0.282\,$ Å), REDUCE (RMSD $= 0.234\,$ Å) and HAAD (RMSD $= 0.208\,$ Å), that were evaluated on the same dataset [9]. This slightly lower accuracy of our algorithm applied to protein structure models compared to the latter two programs may be attributed to the use of a general force field in our work compared to a molecule-specific parametrization. However, the *Universal force field* allows our algorithm to overcome the limitation to a fixed set of molecules.

#### Nucleic acid structures

In the nucleic acid dataset the accuracy is similar to the tested protein structures for fixed hydrogen atoms (RMSD $= 0.13\,$ Å). However, the deviation is significantly larger for polar and nonpolar groups with an RMSD $= 1.20\,$ Å, and RMSD $= 0.71\,$ Å, respectively. Polar hydrogen atoms make up 5.0 % and nonpolar hydrogen atoms make up 1.7 % of all hydrogen atoms in the nucleic acid dataset.

The majority of rotatable polar hydrogen atoms is located at the O2$'$ atoms in RNA molecules. While in experimentally determined structures these hydrogen atoms usually orient toward either

- the O4$'$ atom of the same residue,
- the O3$'$ atom of the same residue or
- the O4$'$ atom of the next residue,

only the latter orientation is selected by the relaxation algorithm.

The rotatable nonpolar hydrogen atoms are bonded to the C7 methyl groups of thymine groups. In the reference structures one hydrogen atom of the methyl groups usually orients toward the O4 oxygen atom. In contrast, the relaxation algorithm chooses various rotamers for the C7 methyl groups, dependent on non-bonded interactions with surrounding atoms. Although the predicted hydrogen atoms are hence not in agreement with the crystallographic results, the output is still plausible: Quantum mechanics calculations show that the maximum energy difference between the rotamers of this methyl group is very low ($1.1\,\text {kcal}\,/\,\text {mol}$) [24] without the influence of ambient atoms.

#### Small molecule structures

Of the 108,502 hydrogen atoms in the small molecule dataset, Hydride was not able to assign 18 of them (0.017 %), since there was no matching fragment in the fragment library. The accuracy for fixed (RMSD $= 0.13\,$ Å) and rotatable nonpolar (RMSD $= 0.27\,$ Å) hydrogen atoms is close to the results from the protein dataset.

Only the rotatable polar hydrogen atoms have a slightly larger deviation (RMSD $= 1.07\,$ Å). In protein structures the orientation of polar hydrogen atoms is often determined by interactions between residues. In case of the small molecule dataset, additional molecules that would favour certain hydrogen orientations are missing, presumably resulting in the observed lower accuracy. To support this assumption, we exemplarily compared the accuracy of predicted rotatable polar hydrogen atoms in a free $\alpha$-d-glucopyranose molecule without chemical environment with predicted atoms in $\alpha$-cyclodextrin bound to a protein receptor (PDB: 5MTU). $\alpha$-cyclodextrin is a 6-mer of $\alpha$-d-glucopyranose monomers. We found an RMSD $= 1.35\,$ Å and RMSD $= 1.15\,$ Å for the free and bound molecule, respectively. Hence, in this case the added chemical environment seemingly contributed to an increase of accuracy.

### Compatibility of fragments for different geometries

As already outlined, the addition of hydrogen atoms does not distinguish between different possible geometries of heavy atoms in a fragment, if the library key is equal. Therefore, we tested whether a fragment library built from fragments with a particular geometry can be used to accurately place hydrogen atoms in fragments with another geometry and vice versa. To this end we investigated the molecule pairs

- $\alpha$-d-glucopyranose and $\alpha$-d-glucofuranose with respect to the C3 atom and
- benzene and cyclobutadiene

(Fig. 2). The respective structures were downloaded from the *PubChem* database.

When the hydrogen position for the C3 atom of $\alpha$-d-glucopyranose (target molecule) is predicted using a library with fragments from $\alpha$-d-glucofuranose (reference molecule), the distance to the original C3 hydrogen atom in $\alpha$-d-glucopyranose is $0.025\,$ Å and $0.070\,$ Å in the opposite case. For the pair benzene and cyclobutadiene the deviation is $0.006\,$ Å for both cases. These deviations are smaller than the mean amplitude of molecular vibration of a C–H bond ($\approx 0.08\,$ Å) [25–27] and hence negligible.

The workflow and scripts to reproduce this benchmark are also included in Additional file 1.

### Computation time

**Fig. 4:**
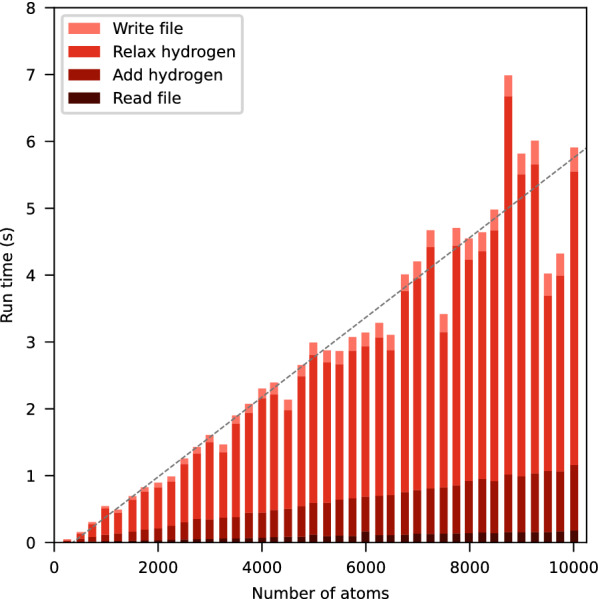
Run time of Hydride. This figure shows the run time of Hydride for different molecular system sizes. Each bar shows the run time (average of 10 executions) for one structure from the PDB. Each run time is divided into the individual computation steps. The gray line represents a linear regression

We measured the time Hydride requires for addition and relaxation of hydrogen atoms to macromolecular structures of different sizes on an *Intel® Core™ i7-8565U* CPU with 8 $\times$ 1.80 GHz (Fig. 4).

The measurements indicate that the run time scales approximately linear with the number of atoms in the molecular system ($R^2 = 0.92$), whereby the relaxation requires the major part. In a naive implementation a quadratic dependence for the relaxation step is expected, as all applicable pairs of atoms need to be evaluated for $V_\text {el}$ and $V_\text {LJ}$. The total number of atom pairs scales quadratically with the number of atoms in the system. However, the implementation uses a cutoff distance of 10 Å, which allows the usage of a cell list to find atom pairs within the cutoff distance in ${\mathcal {O}}(n)$ time complexity instead of ${\mathcal {O}}(n^2)$. Nevertheless, the run time of the relaxation step is additionally dependent on the actual molecular model, since the number of evaluated atom pairs and required iterations to achieve convergence varies for each structure.

The workflow and scripts to reproduce this benchmark are also included in Additional file 1.

## Conclusion

In this work we presented an algorithm that can assign hydrogen atoms to molecular models with accuracy comparable to the methods used by popular software like CHARMM or HAAD. However, these programs require force field parameters for specific molecules, restricting them to molecular structures that contain these molecules exclusively. In contrast, the method presented here is able to assign hydrogen atoms for a wide range of molecular systems. We showed that our technique is able to accurately predict hydrogen positions for almost any organic molecule independent of its size. This advantage is especially convenient when handling biomacromolecules in interaction with ligand molecules.

We bundled our implementation into a documented and easily installable open-source Python package. The CLI offers a fast and straightforward way to add hydrogen atoms to molecular systems, supporting multiple structure file formats for input and output. The Python API on the other hand provides additional flexibility for advanced purposes, such as custom formal or partial charges calculated from other methods or prediction of hydrogen atoms for only a part of the molecular model.

Hence, we think that Hydride and the underlying algorithm is a useful addition to the toolbox of computational and structural biologists.

## Availability and requirements

Project name: Hydride

Project home page: https://hydride.biotite-python.org/

Operating system(s): Windows, OS X, Linux

Programming language: Python

Other requirements: At least Python 3.7, the packages biotite and numpy must be installed

License: BSD 3-Clause

Any restrictions to use by non-academics: None

## Supplementary Information

**Additional file 1**: Accuracy and run time benchmarks.

## Data Availability

The Hydride source code is hosted at https://github.com/biotite-dev/hydride. The official documentation is available at https://hydride.biotite-python.org/. Additionally, the distribution at version 1.1, that was used in the presented benchmarks, is available as archive [28].

## Abbreviations

PDB: Protein Data Bank
CLI: Command line interface
API: Application programming interface
CCD: Chemical component dictionary
UFF: Universal force field
RMSD: Root-mean-square deviation

## References

[1] Berman HM, Westbrook J, Feng Z, Gilliland G, Bhat TN, Weissig H, Shindyalov IN, Bourne PE (2000). The Protein Data Bank. Nucleic Acids Res.

[2] Trott O, Olson AJ (2010). AutoDock Vina: improving the speed and accuracy of docking with a new scoring function, efficient optimization and multithreading. J Comput Chem.

[3] Brooks BR, Bruccoleri RE, Olafson BD, States DJ, Swaminathan S, Karplus M (1983). CHARMM: a program for macromolecular energy, minimization, and dynamics calculations. J Comput Chem.

[4] Webb B, Sali A (2016). Comparative protein structure modeling using MODELLER. Curr Protocols Bioinformat.

[5] Lindahl Abraham, Hess,. van der Spoel. 2021. 10.5281/zenodo.4723561 (GROMACS 2021.2 Manual).

[6] Brooks BR, Brooks CL, MacKerell AD, Nilsson L, Petrella RJ, Roux B, Won Y, Archontis G, Bartels C, Boresch S, Caflisch A, Caves L, Cui Q, Dinner AR, Feig M, Fischer S, Gao J, Hodoscek M, Im W, Kuczera K, Lazaridis T, Ma J, Ovchinnikov V, Paci E, Pastor RW, Post CB, Pu JZ, Schaefer M, Tidor B, Venable RM, Woodcock HL, Wu X, Yang W, York DM, Karplus M (2009). CHARMM: the biomolecular simulation program. J Comput Chem.

[7] Word JM, Lovell SC, Richardson JS, Richardson DC (1999). Asparagine and glutamine: using hydrogen atom contacts in the choice of side-chain amide orientation Edited by J Thornton. J Mol Biol.

[8] O’Boyle NM, Banck M, James CA, Morley C, Vandermeersch T, Hutchison GR (2011). Open Babel: an open chemical toolbox. J Cheminformat.

[9] Li Y, Roy A, Zhang Y (2009). HAAD: a quick algorithm for accurate prediction of hydrogen atoms in protein structures. PLoS ONE.

[10] Kunzmann P, Hamacher K (2018). Biotite: a unifying open source computational biology framework in Python. BMC Bioinformat.

[11] Westbrook JD, Shao C, Feng Z, Zhuravleva M, Velankar S, Young J (2015). The chemical component dictionary: complete descriptions of constituent molecules in experimentally determined 3D macromolecules in the Protein Data Bank. Bioinformatics (Oxford, England).

[12] Kabsch W (1976). A solution for the best rotation to relate two sets of vectors. Acta Crystallogr Sect A.

[13] Kabsch W (1978). A discussion of the solution for the best rotation to relate two sets of vectors. Acta Crystallogr Sect A.

[14] Rappe AK, Casewit CJ, Colwell KS, Goddard WA, Skiff WM (1992). UFF, a full periodic table force field for molecular mechanics and molecular dynamics simulations. J Am Chem Soc.

[15] Ogawa T, Nakano T (2010). The extended universal force field (XUFF): theory and applications. Chem-Bio Informat J.

[16] Gasteiger J, Marsili M (1980). Iterative partial equalization of orbital electronegativity—a rapid access to atomic charges. Tetrahedron.

[17] Lide DR (2003). Handbook of chemistry and physics.

[18] Bradley AR, Rose AS, Pavelka A, Valasatava Y, Duarte JM, Prlić A, Rose PW (2017). MMTF—an efficient file format for the transmission, visualization, and analysis of macromolecular structures. PLOS Comput Biol.

[19] Dalby A, Nourse JG, Hounshell WD, Gushurst AKI, Grier DL, Leland BA, Laufer J (1992). Description of several chemical structure file formats used by computer programs developed at Molecular Design Limited. J Chem Informat Comput Sci.

[20] Harris CR, Millman KJ, van der Walt SJ, Gommers R, Virtanen P, Cournapeau D, Wieser E, Taylor J, Berg S, Smith NJ, Kern R, Picus M, Hoyer S, van Kerkwijk MH, Brett M, Haldane A, del Río JF, Wiebe M, Peterson P, Gérard-Marchant P, Sheppard K, Reddy T, Weckesser W, Abbasi H, Gohlke C, Oliphant TE (2020). Array programming with NumPy. Nature.

[21] Behnel S, Bradshaw R, Citro C, Dalcin L, Seljebotn DS, Smith K (2011). Cython: the best of both worlds. Comput Sci Eng.

[22] Kim S, Chen J, Cheng T, Gindulyte A, He J, He S, Li Q, Shoemaker BA, Thiessen PA, Yu B, Zaslavsky L, Zhang J, Bolton EE (2021). PubChem in 2021: new data content and improved web interfaces. Nucleic Acids Res.

[23] Mölder F, Jablonski KP, Letcher B, Hall MB, Tomkins-Tinch CH, Sochat V, Forster J, Lee S, Twardziok SO, Kanitz A, Wilm A, Holtgrewe M, Rahmann S, Nahnsen S, Köster J. Sustainable data analysis with Snakemake. F1000Research **10**(33) (2021). 10.12688/f1000research.29032.1 10.12688/f1000research.29032.1 PMC8114187 34035898

[24] Mastryukov VS, Fan K, Boggs JE (1995). The effect of methylation on the structure of uracil. J Mol Struct.

[25] Bartell LS, Kuchitsu K, deNeui RJ (1961). Mean and equilibrium molecular structures of methane and deuteromethane as determined by electron diffraction. J Chem Phys.

[26] Cyvin SJ, Cyvin BN, Brunvoll J, Whitmer JC, Klaeboe P, Gustavsen JE (1979). Condensed aromatics. Part III. In-plane molecular vibrations of pyrene. Z Nat A.

[27] Tanimoto M, Kuchitsu K, Morino Y (1971). Molecular structure of diacetylene as studied by gas electron diffraction. Bull Chem Soc Japan.

[28] Kunzmann P, Anter JM. Hydride repository snapshot Zenodo. 2022. 10.5281/zenodo.5997113.

